# LINC01287 regulates tumorigenesis and invasion via miR‐298/MYB in hepatocellular carcinoma

**DOI:** 10.1111/jcmm.13818

**Published:** 2018-08-22

**Authors:** Yichao Mo, Longguang He, Zeru Lai, Zhiheng Wan, Qinshou Chen, Sibo Pan, Liangfu Li, Dasheng Li, Junwei Huang, Fan Xue, Siyao Che

**Affiliations:** ^1^ Department of Hepatobiliary Surgery Gaozhou People's Hospital Gaozhou China; ^2^ Department of General Surgery The First Affiliated Hospital of BaoTou Medical University Inner Mongolia China

**Keywords:** hepatocellular carcinoma, LINC01287, miR‐298, MYB

## Abstract

Recently, it was reported that long non‐coding RNAs (lncRNAs) participated in promoting hepatocellular carcinoma (HCC) initiation and progression. Herein, we reported that the expression level of LINC01287 was elevated in HCC cell lines and tissues. LINC01287 down‐regulation inhibited HCC cells growth and invasion both in vitro and in vivo. LINC01287 exerted as a ceRNA and negatively regulated miR‐298 expression. MYB was identified as a downstream target of miR‐298. The miR‐298/MYB axis mediated LINC01287's effect on HCC. To the best of our knowledge, our findings provided the first evidence that LINC01287 functioned as an oncogene in HCC. LINC01287 may be a candidate prognostic biomarker and a target for new therapies in HCC patients.

## 1 INTRODUCTION

Hepatocellular carcinoma (HCC) is the fifth most common cancer and third leading cause of cancer‐related deaths worldwide.^1^ Many patients that are diagnosed with HCC have already entered the late stage of the disease. Furthermore, metastasis after surgical resection and a high frequency of tumour recurrence contributes to a poor prognosis of HCC patients.^2^ It is therefore important to develop novel strategies for the diagnosis and treatment of HCC.

About 2% of the human genome accounts for protein‐coding genes. The majority of transcripts consists of non‐coding RNAs (ncRNAs)^3^ that can be grouped into the following classes depending on their transcript size: long non‐coding RNAs (lncRNAs) and small ncRNAs.^4^ LncRNAs are transcripts with a length greater than 200 nt. Abnormal expression of lncRNAs often contributes to tumour initiation, growth and metastasis.^5^ A number of articles have demonstrated that lncRNAs are dysregulated in many cancers, including HCC.^6^ Although a great number of lncRNAs have been annotated, the role and molecular regulatory mechanisms of lncRNAs in HCC still need to be further clarified.

MicroRNAs (miRNAs) are a small and single‐stranded RNAs, and they usually negatively regulate the expression of the down‐stream gene via binding at the 3′‐untranslated regions (UTRs).^7^ Mounting documents demonstrate that abnormal expression of miRNAs is involved in tumorigenesis.^8^ Interestingly, literature finds that lncRNAs could function as ceRNAs that compete for miRNA binding, thus derepressing the expression of miRNA‐targeted mRNAs.^9^ The lncRNA‐miRNA‐mRNA regulatory network is suggested to play a critical role in cancer tumorigenesis and progression.^10^, ^11^

We explored the role and underlying mechanism of LINC01287 in HCC. We also explore the interaction between LINC01287 and miR‐298.

## 2 MATERIALS AND METHODS

### 2.1 Cell culture and HCC patients sample collection

Hepatocellular carcinoma cell lines (HepG‐2, Huh7, Bel7402 and Hep3B) and normal liver epithelial cell line LO2 were purchased from the Institute of Biochemistry and Cell Biology of the Chinese Academy of Sciences (Shanghai, China). All cell lines were maintained at 37°C in a humidified 5% CO_2_ atmosphere in PRMI‐1640 medium supplemented with 10% foetal bovine serum.

Hepatocellular carcinoma and matched‐normal tissue samples (formalin‐fixed and embedded in paraffin) were obtained from patients of the Gaozhou People's Hospital. Written informed consent was obtained from all patients, and the project was approved by the Ethical and Scientific Committees of Gaozhou People's Hospital. These enrolled patients were diagnosed as HCC in our hospital between September 2011 and October 2016. The median age of these patients was 54.8 years old. All experimental protocols were approved by the Clinical Research Ethics Committees of Gaozhou People's Hospital. The method to confirm lymph node involvement in HCC patients was carried out as previously described.^12^

### 2.2 Cell transfection, lentivirus production and transduction

The cell transfections were carried out using lipofectamine 2000 reagent (Invitrogen, Carlsbad, USA) according to manufactures instructions. siRNA against MYB (Ruibio, Guangzhou, China) and a non‐targeting siRNA control (Ruibio) were used to knockdown gene expression. The vector pcDNA3.1‐MYB and pcDNA3.1 were purchased from Santa Cruz (Santa Cruz, CA, USA). miR‐298, miR‐ctrl, anti‐miR‐ctrl and anti‐miR‐298 were purchased from Genechem (Shanghai, China).

shRNA sequences targeting LINC01287 were AAGCATTGTAGACCTGGCTGCTGAA. The sequences were cloned into the pGFP‐C‐shLenti vector according to manufacturer's instructions (Origene, Rockville, MD, USA). Then, the viruses were packaged using 293T cells according to standard protocol. HCC cells were infected with virus particles plus 6 μg/mL Polybrene (Sigma‐Aldrich, St. Louis, MO, USA).

### 2.3 Quantitative real‐time PCR assay

Total RNA was extracted from HCC cells using TRIzol reagent (Invitrogen) according to the manufacturer's instructions. The RNA was reverse transcribed to cDNA, followed by real‐time PCR analyses. The primers used in the study were listed in Table 1.

**Table 1:** Primers used in the study

	Forward: 5′‐3′	Reward: 3′‐5′
GAPDH	TCAAGATCATCAGCAATGCC	CGATACCAAAGTTGTCATGGA
U6	ATACAGAGAAAGTTAGCACGG	GGAATGCTTCAAAGAGTTGTG
LINC01287	GGTTGATGTAAGGACCTCGT	GAGACCTTGTTTCATGTGTCG
MIR‐298	TCAGGTCTTCAGCAGAAGC	TAGTTCCTCACAGTCAAGGA
MYB	CGCACTTTAGATTCATTGATGC	AGGTGAGGGACTCAAACTG
LINC01287 P1	CGAGTACTTCTAAATCCCAGT	AAAGGGTTCTCTCACTAAAAG
LINC01287 P2	AGAAATCTATATTGACAGT	CCTGGGTAGGAATTGTAAGCGA
LINC01287 P3	ATAGGCTGAAATGCACTGAAC	GACAAAAACCGCAGCGAGCGG
LINC01287 P4	TCCGTGTGTGGTGGTACTGG	GATTAAAACATAAAAATCAT

To determine the subcellular distribution of LINC01287, nuclear and cytoplasm fractions of cells were separated using the PARIS Kit (Life Technologies, Grand Island, NY, USA) according to the manufacturer's instructions. Then, the RNA was extracted from both fractions. Subsequently, real‐time PCR assay was used to examine the expression ratios of specific RNA molecules between the nuclear and cytoplasm fractions.

### 2.4 MTT, colony formation and cell cycle assays

For MTT assay, HCC cells were seeded in 96‐well plates. After 24 hours, 5 mg/mL MTT was added to each well. The HCC cells were then incubated for another 6 hours. Subsequently, DMSO was added into each well and the absorbance was read at 490 nm under spectrophotometer.

For clone formation assay, HCC cells were seeded in 6‐well culture plates. Two weeks later, the cells were fixed with paraformaldehyde, stained with haematoxylin solution and counted under a microscope.

For cell cycle assay, cells were harvested from culture dishes and washed three times by cold PBS. Then, the cells were fixed with 70% ice‐cold ethanol at 4°C overnight. Finally, the cells were incubated with propidium iodide (supplemented with RNase A). The FACS calibre flow cytometry (BD Biosciences, Franklin Lakes, NJ, USA) was used to examine the DNA content of labelled cells.

### 2.5 Boyden assay

For Boyden assay, HCC cells in serum‐free DMEM were seeded in upper chambers, which were inserted into a 24‐well plate. PRMI‐1640 supplemented with 10% FBS were added into the lower chamber of each well. Twenty‐four hours later, HCC cells that stayed on the upper surface of the membrane were removed and the cells that migrated to the lower membrane were fixed with paraformaldehyde, stained by crystal violet and counted.

### 2.6 Western blot assay and immunofluorescence assay

For Western blot assay, the protein samples were transferred onto an PVDF membrane, followed by incubation for overnight in a 1:500 dilution of primary antibodies in 4°C. Subsequently, the membrane was incubated with HRP‐conjugated rabbit or mouse for 1 hour at room temperature and then developed with a chemiluminescence reagent.

For immunofluorescence assay, cells were plated on culture slides. Twenty‐four hours later when cells were attached to the culture slides, cells were rinsed with phosphate‐buffered saline (PBS) three times and then were fixed in ice‐cold methanol‐acetone for 10 minutes. Subsequently, cells were blocked for 10 minutes in 5% BSA in PBS, followed by incubated with primary antibodies in PBS for 1.5 hours at room temperature. After washes with PBS three times, the slides were incubated for 40 minutes with secondary antibodies. After three washes, the slides were stained with 4‐,6‐diamidino‐2‐phenylindole (DAPI) for 10 minutes and were examined using an Olympus confocal imaging system.

### 2.7 In situ hybridization assay

In situ detection of lncRNA was performed as previously described.^13^

### 2.8 Luciferase reporter assay

We cloned the full‐length MYB cDNA (lacking the 3′‐UTR) into the eukaryotic expression vector pcDNA3.1 (Invitrogen). Subsequently, the 3′‐UTR untranslated region of MYB was amplified and cloned downstream of the firefly luciferase gene in the pGL3 vector (Promega, Madison, WI, USA) and the vector as named wild‐type (WT) MYB‐3′‐UTR. Using GeneTailor Site‐Directed Mutagenesis System (Invitrogen), we made site‐directed mutagenesis of the miR‐298 binding sites in the MYB 3′‐UTR. The vector was named mutant type (MUT) MYB‐3′‐UTR. Subsequently, we cotransfected the HCC cells with the wt or mut MYB‐3′‐UTR vector and miR‐298 mimic or inhibitor. Finally, we performed the luciferase assay using the Dual‐Luciferase Reporter Assay System (Promega) 36 hours after transfection. To perform LINC01287 promoter luciferase assays, HCC cells were seeded into 24‐well plates and cotransfected with plasmids that contain LINC01287 promoter, the pRL‐TK‐Renilla plasmid (Promega) and pcDNA.3‐c‐jun.

### 2.9 In vivo tumour growth and invasion assay

All procedures involving animals were approved by the Institutional Committee on Animal Care at Gaozhou People Hospital. Huh7 cells were injected subcutaneously into both flanks of nude mice. Three mice were used in each group. Four weeks after implantation, the xenografts were removed from the mice and weighed. The tumour volume was calculated using the following formula: 4π/3 × (width/2)^2^ × (length/2). Huh7 cells were used for invasion assay in vivo. The invasion assay was performed as previously described.^14^

### 2.10 Statistical analysis

SPSS 13.0 (IBM, New York, USA) and GraphPad Prism 5.0 (California, USA) softwares were used for statistical analysis. The values are shown as the mean ± SEM. Analyses of different groups were performed using one‐way ANOVA or two‐tailed Student's *t* test. *P* < 0.05 was considered statistically significant.

## 3 RESULTS

### 3.1 Detection of LINC01287 expression in HCC tissues along with cell lines

**Figure 1:**
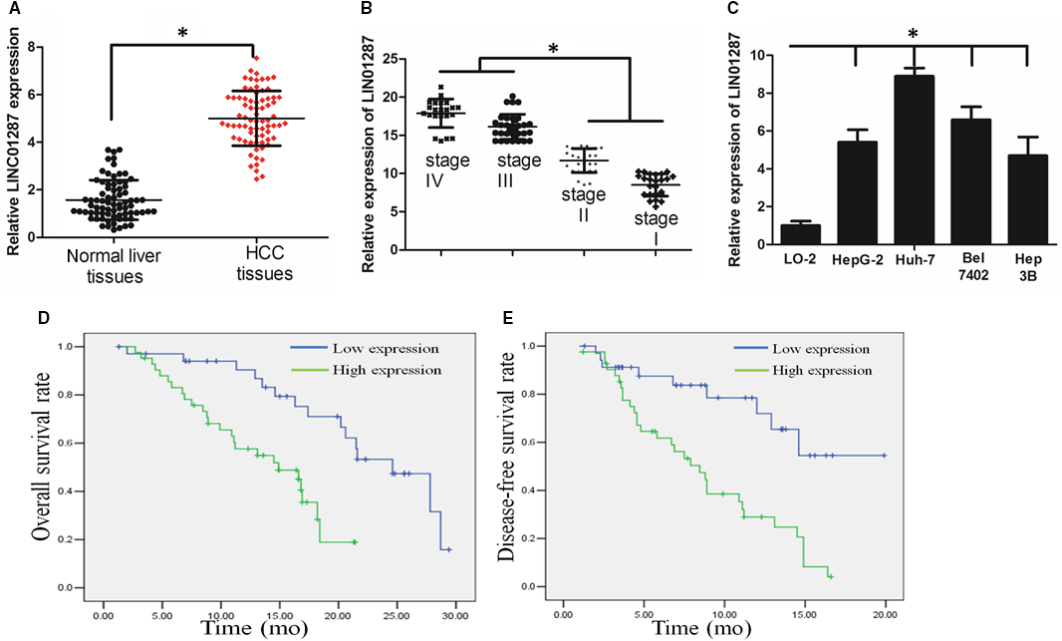
LINC01287 expression level was up‐regulated in hepatocellular carcinoma (HCC) cell lines and tissues. A, LINC01287 expression was significantly increased in primary HCC tissues. B, The expression level of LINC01287 was up‐regulated in advanced stage HCC patients. C, LINC01287 expression was higher in HCC cells when compared with a normal liver cell line. D and E, High‐level expression of LINC01287 was associated with a shorter overall survival and disease‐free survival time of HCC patients (blue and green curves represent low expression and high expression of LINC01287, respectively). *Represents *P* value < 0.05

We focused a list of lncRNAs that were significantly elevated in HCC samples using online software program circlncRNAnet.^15^ Because the expression level of LINC01287 was the highest among these lncRNAs and the function of LINC01287 has not been explored in HCC, we chose it for further study (Table S1). Subsequently, we examined the expression level of LINC01287 in 117 HCC patients. It was revealed that LINC01287 was up‐regulated in HCC tissues, when compared with normal tissues (Figure 1A). In addition, LINC01287 was up‐regulated in late‐stage HCC patients (Figure 1B). In consistence, LINC01287 was elevated in HCC cell lines, compared with that in the normal human liver cell line LO2 (Figure 1C). In addition, we confirmed that LINC01287 was up‐regulated in HCC tissues when compared with the normal tissues using TCGA database (Figure S1C,D). LINC01287 was mainly located in the cytoplasm, as determined by subcellular fractionation and real‐time PCR assays (Figure S1A). The in situ hybridization assay further confirmed that LINC01287 was mainly located in the cytoplasm (Figure S1B).

We analysed the relation between LINC01287 expression level and patients‧ clinicopathological features in Table 2. The age, gender and HBV infection did not associate with LINC01287 expression level. Nevertheless, high LINC01287 expression was significantly associated with tumour size (*P* = 0.004), lymph node metastasis (*P* = 0.011) and late clinical stage (*P* = 0.046). HCC patients with high expression level of LINC01287 had poorer overall survival rate and disease‐free rate, as revealed by Kaplan‐Meier assay (Figure 1D and E, *P* < 0.05).

**Table 2:** Associations between lncRNA LINC01287 expression and patients‧ clinicopathological features

Variable	No. of patients	LINC01287 low expression	LINC01287 high expression	*P* value
Age				
<60	70	38	32	0.308
≧60	47	21	26	
Gender				
Male	69	36	33	0.548
Female	48	23	25	
Tumour size				
<5 cm	60	38	22	0.004
≧5cm	57	21	36	
Lymph node involvement				
Absent (pN0)	50	32	18	0.011
Present (pN+)	67	27	40	
TNM stage				
I‐II	47	29	18	0.046
III‐IV	70	30	40	
HBV infection				
Yes	54	32	22	0.077
No	63	27	36	

### 3.2 LINC01287 inhibition decreased HCC cell proliferation and invasion

**Figure 2:**
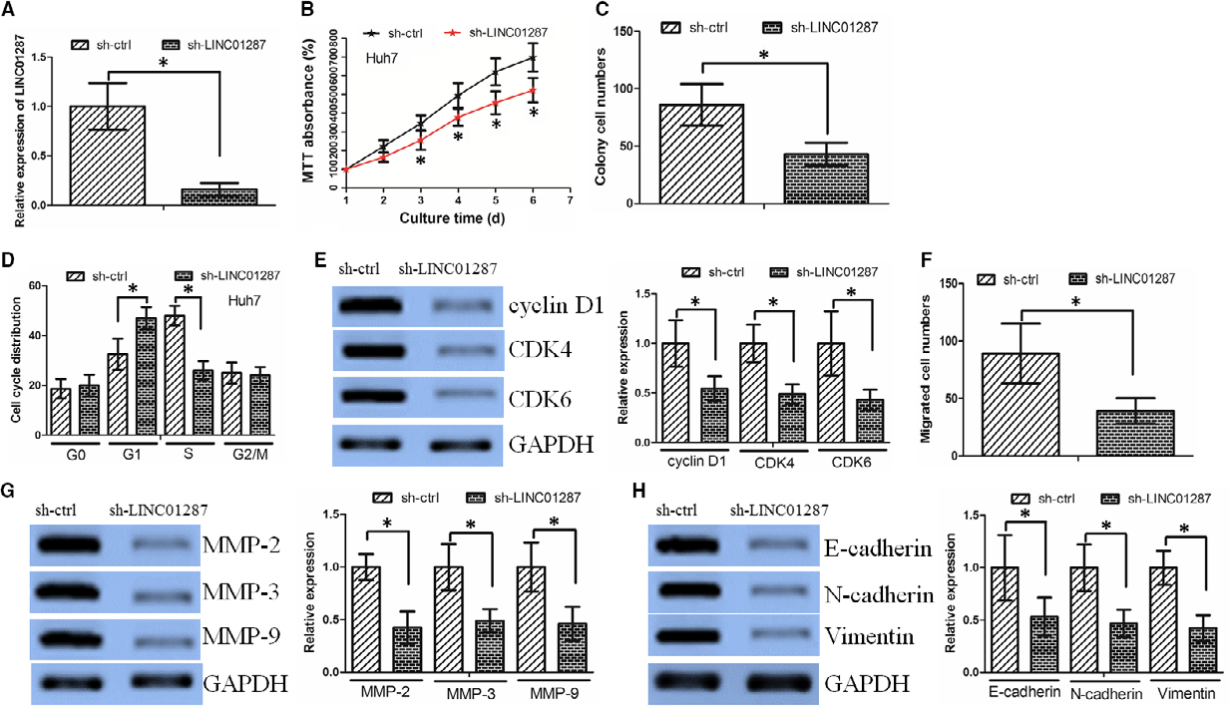
LINC01287 inhibition decreased hepatocellular carcinoma (HCC) cell proliferation and invasion. A, LINC01287 expression in Huh‐7 cells transduced with control shRNA vector (sh‐ctrl) or LINC01287 shRNA vector (sh‐LINC01287). B, The MTT assay revealed that LINC01287 down‐regulation significantly decreased cell proliferation. C, Colony formation assay demonstrated that oncogenic survival was significantly decreased in sh‐LINC01287 cells when compared with sh‐ctrl cells. D, sh‐LINC01287 cells displayed a significantly higher frequency of cells at the G1 phase and a lower frequency of cells at S phase. E, LINC01287 down‐regulation affected the expression of G1/S phase checkpoint proteins. F, LINC01287 down‐regulation decreased the HCC cell invasion ability, as revealed by the Boyden assay. G, The expression levels of MMP‐2, MMP‐3 and MMP‐9 protein were lower in sh‐LINC01287 cells. H, Expression level of E‐cadherin increased, while expression levels of N‐cadherin and vimentin were decreased in sh‐LINC01287 cells

We chose Huh7 cell line (which had the highest expression of LINC01287) for further study. Huh7 cells that stably expressed low expression level of LINC01287 (sh‐LINC01287) were established (Figure 2A). LINC01287 inhibition significantly decreased cell proliferation, as determined by MTT assay (Figure 2B). In consistence, LINC01287 inhibition impaired colony formation ability (Figures 2C and S2A). sh‐LINC01287 cells demonstrated a significantly higher frequency of cells in the G1 phase and a lower frequency of cells in the S phase, as revealed by flow cytometry assay (Figures 2D and S2B). Western blot assay revealed that the expression of G1/S phase checkpoint proteins such as cyclin D1, CDK4 and CDK6 was significantly decreased in sh‐LINC01287 cells (Figure 2E).

We further asked whether LINC01287 affected cell invasion in HCC. Inhibition of LINC01287 decreased HCC cell invasion, as revealed by Boyden assay (Figures 2F and S2D). In addition, inhibition of LINC01287 decreased MMP‐2, MMP‐3 and MMP‐9 expression (Figure 2G). E‐cadherin expression was elevated, while N‐cadherin expression and vimentin expression were down‐regulated in sh‐LINC01287 cells, compared to sh‐ctrl cells (Figure 2H).

### 3.3 LncRNA LINC01287 negatively regulated miR‐298 expression

**Figure 3:**
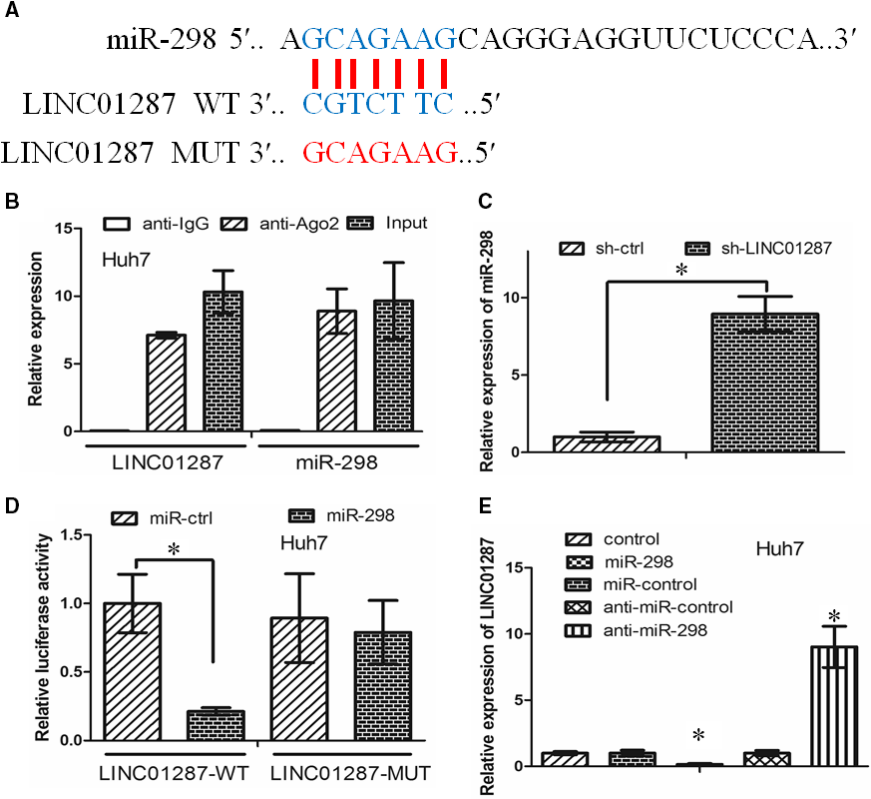
LncRNA LINC01287 acted as ceRNA to regulate miR‐298 expression. A, The binding sites of miR‐298 on LINC01287. B, The RIP assay revealed that LINC01287 and miR‐298 were enriched in the same Ago2 immunoprecipitates. C, MiR‐298 expression was increased in sh‐LINC01287 cells when compared with sh‐ctrl cells. D, Cotransfection of miR‐298 and LINC01287‐Wt strongly decreased the luciferase activity, while cotransfection of miR‐ctrl and LINC01287‐Wt did not change the luciferase activity. Cotransfection of miR‐298 and LINC01287‐Mut did not change the luciferase activity either. E, miR‐298 decreased LINC01287 expression, while anti‐miR‐298 increased LINC01287 expression

LncRNAs usually exerted their function via acting as a sponge of miRNAs.^16^ We thus asked whether there was an interaction between LINC01287 and miRNAs in HCC. We identified several microRNAs that may be regulated by LINC01287 via the online software miRDB (http://mirdb.org/miRDB/index.html) (Figure S3A). We examined the different expression of these microRNAs between sh‐ctrl and sh‐LINC01287 group in Huh7 cells. It was found that three microRNAs (miR‐298, miR‐4308 and miR‐23c) altered significantly between sh‐ctrl and sh‐LINC01287 group (Figure S3B). The binding sites of miR‐298 on LINC01287 were indicated in Figure 3A. MiRNAs directed their downstream targets through a ribonucleoprotein complex, which was called the RNA‐induced silencing complex (RISC), the core component of which was Ago2.^17^ RNA immunoprecipitation (RIP) assay was performed to examine whether LINC01287 and miR‐298 were in the same RISC complex. We found that LINC01287 and miR‐298 were enriched in Ago2 immunoprecipitates when compared to control IgG immunoprecipitates (Figure 3B, *P* < 0.05). When LINC01287 was inhibited, the expression level of miR‐298 was elevated in Huh7 cells (Figure 3C, *P* < 0.05). Next, we tested whether miR‐298 inhibited LINC01287 expression. The predicted miR‐298 binding site of LINC01287 (LINC01287‐WT), together with a mutated miR‐298 binding site of LINC01287 (LINC01287‐MUT), was cloned into a reporter plasmid. The luciferase activity in Huh7 cells cotransfected with miR‐298 and LINC01287 decreased. However, the luciferase activity in Huh7 cells cotransfected with miR‐ctrl and LINC01287‐WT or cotransfected with miR‐298 and LINC01287‐MUT did not change significantly (Figure 3D, *P* < 0.05). Subsequently, we asked whether miR‐298 inhibited LINC01287 expression. It was found that miR‐298 decreased LINC01287 expression, compared to miR‐ctrl treatment. Nevertheless, anti‐miR‐298 elevated LINC01287 expression, compared to anti‐miR‐ctrl treatment (Figure 3E, *P* < 0.05). In all, our data demonstrated that there was reciprocal interaction between LINC01287 and miR‐298.

The biological role of miR‐298 was the investigated in HCC. The expression level of miR‐298 was down‐regulated in HCC cell lines and tissues, when compared with LO2 cell line and normal liver tissues, respectively (Figure S3C,D). miR‐298 decreased HCC cell growth and invasion (Figure S3E‐G). In all, our data revealed that miR‐298 functioned as a tumour suppressor.

### 3.4 LINC01287 regulated MYB expression through miR‐298

**Figure 4:**
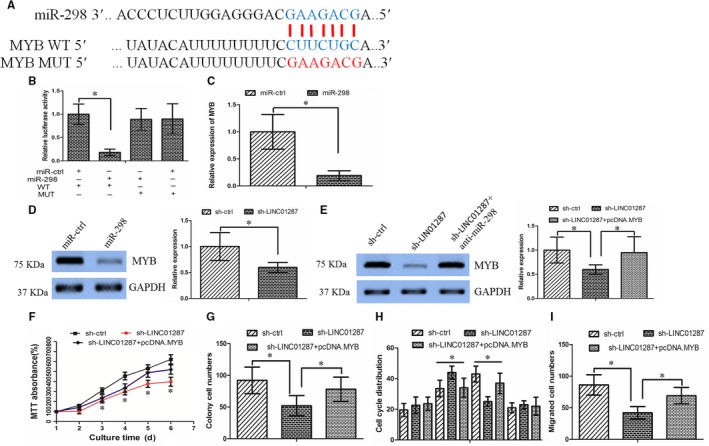
MYB was a downstream target of miR‐298. A, The binding sites of miR‐298 on MYB. B, The luciferase assay showed that cells transfected with miR‐298 had less luciferase activity than those transfected with miR‐ctrl. C, miR‐298 repressed MYB mRNA expression in hepatocellular carcinoma (HCC) cells. D, miR‐298 repressed MYB protein expression in HCC cells. E, Anti‐miR‐298 treatment led to the restoration of MYB in sh‐LINC01287 cells. F, The MTT assay revealed that sh‐LINC01287 cells grew more slowly than the sh‐ctrl cells, while overexpression of MYB rescued the effect. G, The colony formation assay showed that sh‐LINC01287 cells formed smaller and fewer colonies than the sh‐ctrl cells, which was counteracted by overexpression of MYB. H, LINC01287 down‐regulation affected the cell cycle distribution, which was counteracted by overexpression of MYB. I, LINC01287 down‐regulation inhibited HCC cell invasion ability, which was rescued by overexpression of MYB

We searched the potential targets of miR‐298 using software TargetScan. These potential downstream genes were related to cell differentiation, proliferation, migration and apoptosis (Figure S3H), as revealed by Kyoto Encyclopedia of Genes and Genomes and Gene oncology assays. MYB regulated cancer proliferation and invasion in HCC, and we chose it for further study. As indicated in Figure 4A, we cloned wild‐type 3′‐UTR region of MYB mRNA (including the predicted miR‐298 recognition site) and the mutant type (excluded the predicted miR‐298 recognition site into luciferase reporter plasmids). We observed that miR‐298 decreased luciferase activity in the wild‐type vector, but not that in the mutant type (Figure 4B). Interestingly, both mRNA and protein expression of MYB was decreased by miR‐298 treatment (Figure 4C and D). In summary, these findings demonstrated that MYB was a downstream target of miR‐298.

Next, we examined whether the miR‐298/MYB axis mediated LINC01287's function. Western blot assay revealed that down‐regulation of LINC01287 inhibited MYB expression. Nevertheless, LINC01287 down‐regulation effect on MYB expression was abolished in the case of miR‐298 inhibition (Figure 4E). Interestingly, restoration of MYB could counteract LINC01287 down‐regulation's effect on cell growth, cell cycle distribution and invasion (Figure 4F‐I).

In all, our findings revealed that LINC01287 could exert its function through the miR‐298/MYB axis.

### 3.5 LINC01287 inhibition decreased tumour growth in vivo

**Figure 5:**
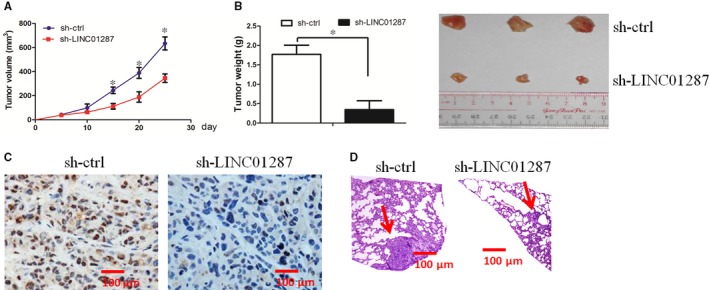
LINC01287 inhibition decreased tumour growth and invasion in vivo. A, Compared with sh‐ctrl cell‐derived xenograft tumours, sh‐LINC01287 cell‐derived xenograft tumours grew more slowly. B, The mean weight of sh‐LINC01287 cell‐derived xenograft tumours was significantly less when compared with sh‐ctrl cell‐derived xenograft tumours. C, Knockdown of LINC01287 significantly decreased the percentage of Ki‐67‐positive cells in tumours when compared with the negative control group. D, Knockdown of LINC01287 decreased lung metastasis in vivo

The effect of LINC01287 on tumour growth in vivo was tested. HCC cell tumours grew more slowly when LINC01287 was inhibited (Figure 5A). Compared with sh‐ctrl group, the mean weight of xenograft tumours was less in the sh‐LINC01287 group (Figure 5B). The Ki‐67 staining assay revealed that the proliferation index was lower in the sh‐LINC01287 group, compared with sh‐ctrl group (Figure 5C). Finally, LINC01287 down‐regulation inhibited lung metastasis ability in vivo (Figure 5D). In all, we revealed that LINC01287 promoted tumour growth and invasion in vivo.

## 4 DISCUSSION

Recently, the role and the underlying mechanism of lncRNAs in cancer have been well documented.^18^ Targeting the abnormal expression of lncRNAs may be effective in cancer treatment.

In the current study, we revealed that LINC01287 was up‐regulated in HCC cell lines and tissues. LINC01287 down‐regulation decreased cell proliferation, together with colony formation ability in vitro. In consistence, LINC01287 down‐regulation decreased tumour growth in vivo. LINC01287 down‐regulation contributed to cell cycle arrest in G1 stage. The G1/S phase checkpoint proteins (eg c‐myc, cyclin D1 and CDK4) were altered when LINC01287 was inhibited. These data suggested that LINC01287 may lead to cell growth via affecting cell cycle progress. We also revealed that LINC01287 down‐regulation inhibited GC cell invasion in vitro and decreased lung metastasis in vivo. The EMT plays vital role in promoting cancer cell invasion.^19^ We thus asked whether LINC01287 was involved in EMT phenotype. It was revealed that LINC01287 down‐regulation increased epithelial marker E‐cadherin while decreased mesenchymal markers N‐cadherin and vimentin expression. These data suggested that LINC01287 may promote EMT phenotype and thus lead to HCC cell invasion.

Previous study revealed that lncRNAs may act as endogenous molecular sponges to compete for miRNAs and negatively regulating miRNA expression.^20^, ^21^ We identified several miRNAs that may interact with LINC01287 using online software. Among these miRNAs, we chose miR‐298 for further study, as the level of miR‐298 expression was significantly increased in sh‐LINC01287 cells. The role of miR‐298 was seldom reported in HCC. We revealed that miR‐298 may be a tumour suppressor in HCC. We confirmed the regulating relationship between LINC01287 and miR‐298 as following data: (a) LINC01287 down‐regulation increased miR‐298 expression; (b) the luciferase activity assay confirmed the direct binding ability of the predicted miR‐298 binding site on LINC01287; (c) the RIP assays found that LINC01287 and miR‐298 were in the same RISC.

Emerging evidence demonstrated the role of MYB in cancer progression, invasion and metastasis.^22^ MYB was identified as a downstream target of miR‐298 in our study. Our data revealed that LINC01287 inhibition decreased MYB expression. However, when miR‐298 was inhibited, LINC01287 down‐regulation effect on MYB expression was abolished. Overexpression of MYB could counteract LINC01287's effect on HCC cells. These data suggested that LINC01287 exerted its function through the miR‐298/MYB axis. We further confirmed that there was a positive correlation between MYB and LINC01287 in the HCC tissues. Taken together, our findings revealed that there was feedback loop between LINC01287/miR‐298/MYB axis.

In all, our data provided the first evidence that the LINC01287/miR‐298/MYB axis controlled cell growth and invasion in HCC cells. Therapeutics that target LINC01287 may improve the treatment of HCC.

## 5 CONCLUSIONS

In conclusion, our study demonstrated that LINC01287 was up‐regulated in HCC and may function as a ceRNA to increase MYB expression by sponging miR‐298, which consequently contributed to HCC growth and metastasis. Our findings indicated that LINC01287 could be a potential therapeutic target for HCC treatment.

## COMPETING INTERESTS

The authors declare that they have no competing interests.

## AUTHORS‧ CONTRIBUTIONS

Siyao Che and Yichao Mo performed the study concept and design; Longguang He, Zeru Lai and Zhiheng Wan involved in acquisition of data; Qinshou Chen, Sibo Pan, Liangfu Li and Dasheng Li involved in analysis and interpretation of data; Junwei Huang1 and Fan Xue drafted the manuscript. All authors read and approved the final manuscript.

## ETHICS APPROVAL AND CONSENT TO PARTICIPATE

The study on HCC cancer samples was approved and supervised by the Research Ethics Committee of Gaozhou people's Hospital. Written informed consents were obtained from all patients. The animal experiments were performed in strict accordance with the guidelines of the Research Animal Care Committee Gaozhou people's Hospital.
