# Supplementary figures and images for: LINC01287 regulates tumorigenesis and invasion via miR‐298/MYB in hepatocellular carcinoma
Source: J Cell Mol Med. 2018 Aug 22;22(11):5477–85. doi: 10.1111/jcmm.13818 (PMC6201358; doi:10.1111/jcmm.13818)

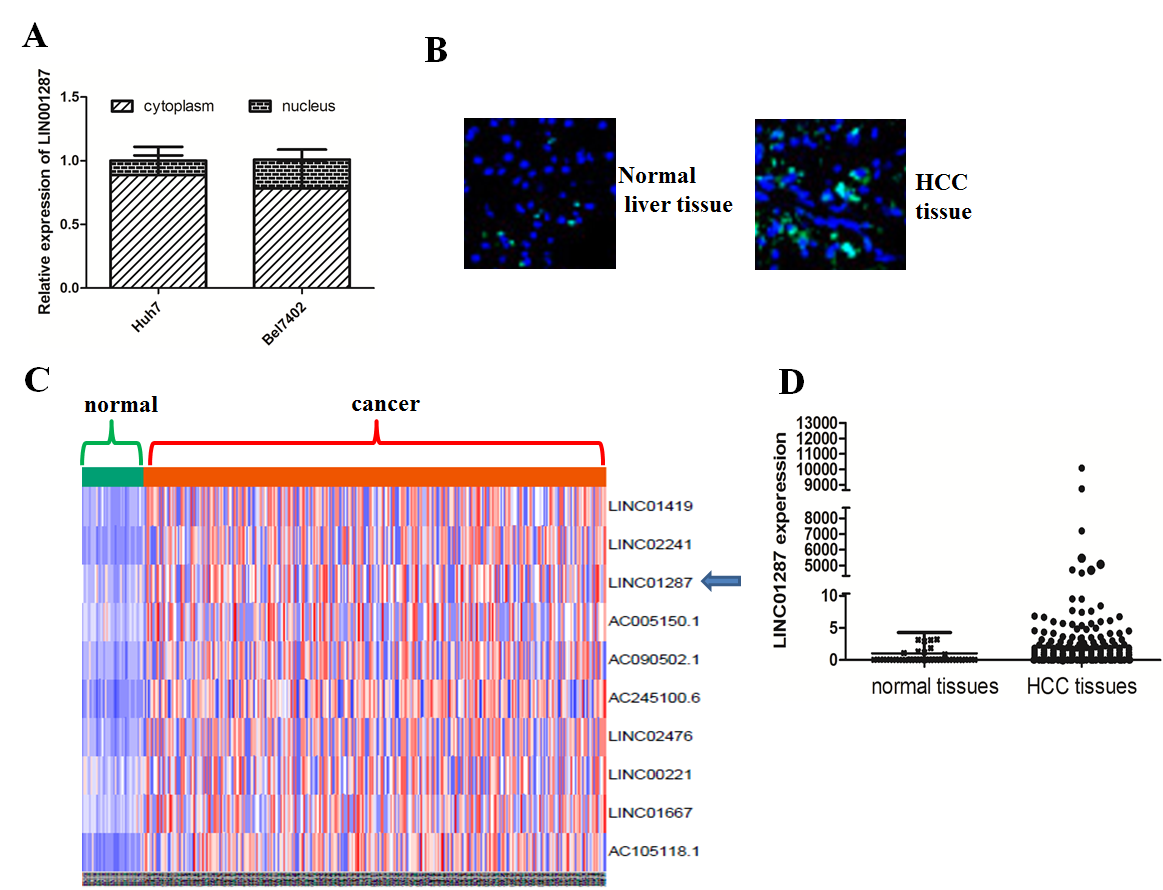

Supplement: Supplementary file 1 [file JCMM-22-5477-s001.tif]

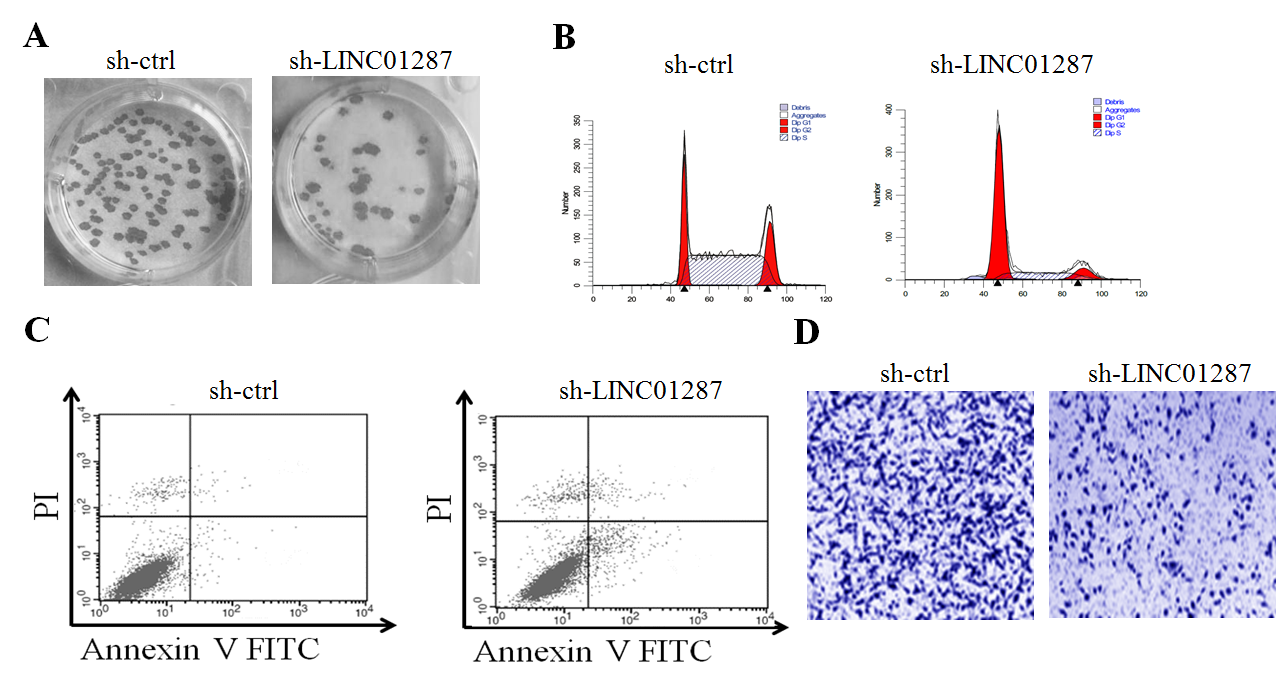

Supplement: Supplementary file 2 [file JCMM-22-5477-s002.tif]

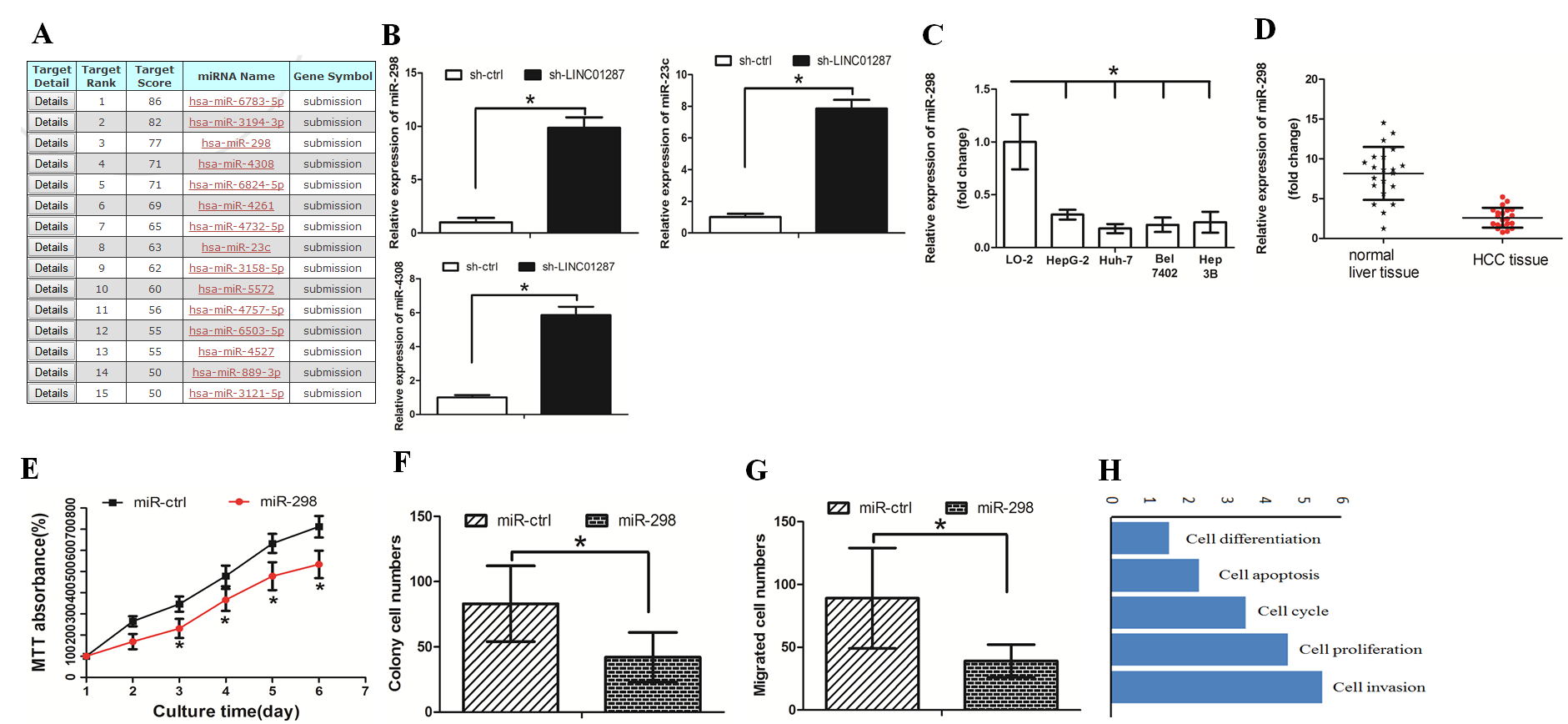

Supplement: Supplementary file 3 [file JCMM-22-5477-s003.tif]
